# Development and validation of nomogram based on lncRNA ZFAS1 for predicting survival in lymph node-negative esophageal squamous cell carcinoma patients

**DOI:** 10.18632/oncotarget.19937

**Published:** 2017-08-04

**Authors:** Hongtai Shi, Zhenhua Liu, Dong Pei, Youqin Jiang, Haiwen Zhu, Bin Chen

**Affiliations:** ^1^ Department of Radiotherapy, The Third People's Hospital of Yancheng, Yancheng 224005, China; ^2^ Department of Radiotherapy, Yancheng City No.1 People's Hospital, Yancheng 224000, China

**Keywords:** lncRNA, ZFAS1, nomogram, ESCC

## Abstract

**Background:**

There is increasing evidence of a relationship between long non-coding RNA (lncRNA) and cancer. This study aimed to examine the prognostic value of the lncRNA ZFAS1 in esophageal squamous cell carcinoma (ESCC).

**Results:**

The results showed that ZFAS1 expression was significantly higher in ESCC tissues compared with the corresponding adjacent normal tissues (*P* < 0.001). ESCC patients with high ZFAS1 expression had a poor overall survival (OS). Histological grade, T stage and ZFAS1 expression were integrated to develop the nomogram. The nomogram showed a significantly better prediction of OS for patients with lymph node-negative ESCC. The ROC curve also showed higher specificity and sensitivity for predicting 3- and 5-year ESCC patient survival compared with the AJCC staging system. The decision curve analysis also indicated a greater potential for the nomogram in clinical application compared with the AJCC staging system. Importantly, our findings were supported by a validation cohort.

**Materials and Methods:**

We retrospectively investigated 398 lymph node-negative ESCC patients. Data from the primary cohort (*n* = 246) were used to develop a multivariate nomogram. The nomogram was internally validated for discrimination and calibration with bootstrap samples and was externally validated with an independent patient cohort (*n* = 152).

**Conclusions:**

Our proposed nomogram, which integrates clinicopathological factors and ZFAS1 expression, can accurately predict the prognosis of lymph node-negative ESCC patients without preoperative chemoradiotherapy.

## INTRODUCTION

Esophageal cancer is a common malignant tumor of the digestive tract and has the characteristics of a gradual onset, rapid progress, and poor prognosis. There are nearly 330,000 new cases each year in the world, and there are more than 270,000 deaths from esophageal cancer each year [1]. The disease is particularly prevalent in China, with the predominant subtype being esophageal squamous cell carcinoma (ESCC), which ranks in the top four for the highest mortality rates for malignant tumors [2]. Over the past decade, the mortality rate has significantly decreased as a result of improved diagnostic tools and better treatment options. Nonetheless, the 5-year survival rate remains low [3]. Tumor-Node-Metastasis (TNM) staging is one of the most important prognostic factors used to determine therapeutic strategies and to predict therapeutic response. However, the prognostic value of TNM staging for esophageal cancer is not satisfactory. Therefore, there is an urgent need to discover new biomarkers that can predict long-term survival and identify therapeutic strategies for ESCC.

Emerging evidence has found that long non-coding RNA (lncRNA), defined as non-protein coding RNAs longer than 200 nucleotides, participates in the occurrence and development of many human diseases by regulating gene expression at the transcriptional, post-transcriptional and epigenetic levels [4–6]. Moreover, recent reports have demonstrated a relationship between lncRNA and cancer [7, 8], suggesting that lncRNA may be important for tumor development.

Zinc finger antisense 1 (ZFAS1) is a newly discovered lncRNA [9]. It is located on chromosome 20q13, which is the antisense strand of ZNFX1 and the carrier of SNORD12C, SNORD12B and SNORD12 [9]. Recent evidence has indicated that ZFAS1 plays an important role in malignancies [10–22]. For example, altered expression of ZFAS1 has been reported in breast [9, 11], liver [10], gastric [13, 15, 21], lung [17], glioma [18, 22], ovarian [19, 20] and colorectal cancers [12, 14, 16]. Moreover, the expression of ZFAS1 has been found to correlate with patient prognosis in lung [17], colorectal [14], and gastric cancers [15] as well as glioma [18]. However, the prognostic value of ZFAS1 in esophageal cancer has not yet been reported. The objective of this study was to identify whether ZFAS1 was differentially expressed between ESCC and para-tumorous tissues and to investigate whether there was a correlation between ZFAS1 expression and patient prognosis or other clinicopathological parameters. We aimed to use this information to establish a nomogram for ZFAS1 and clinicopathological parameters to provide a more accurate tool for assessing ESCC prognosis. Advanced esophageal cancer patients often need preoperative neoadjuvant chemoradiotherapy, which may affect changes in lncRNA in the surgical specimen. Therefore, only ESCC patients without preoperative chemotherapy were selected in this study, and they were mainly lymph node-negative, which meant that lymph node-negative ESCC patients without preoperative chemoradiotherapy were the final selection.

## RESULTS

### lncRNA ZFAS1 expression was up-regulated in ESCC tissues

**Figure 1:**
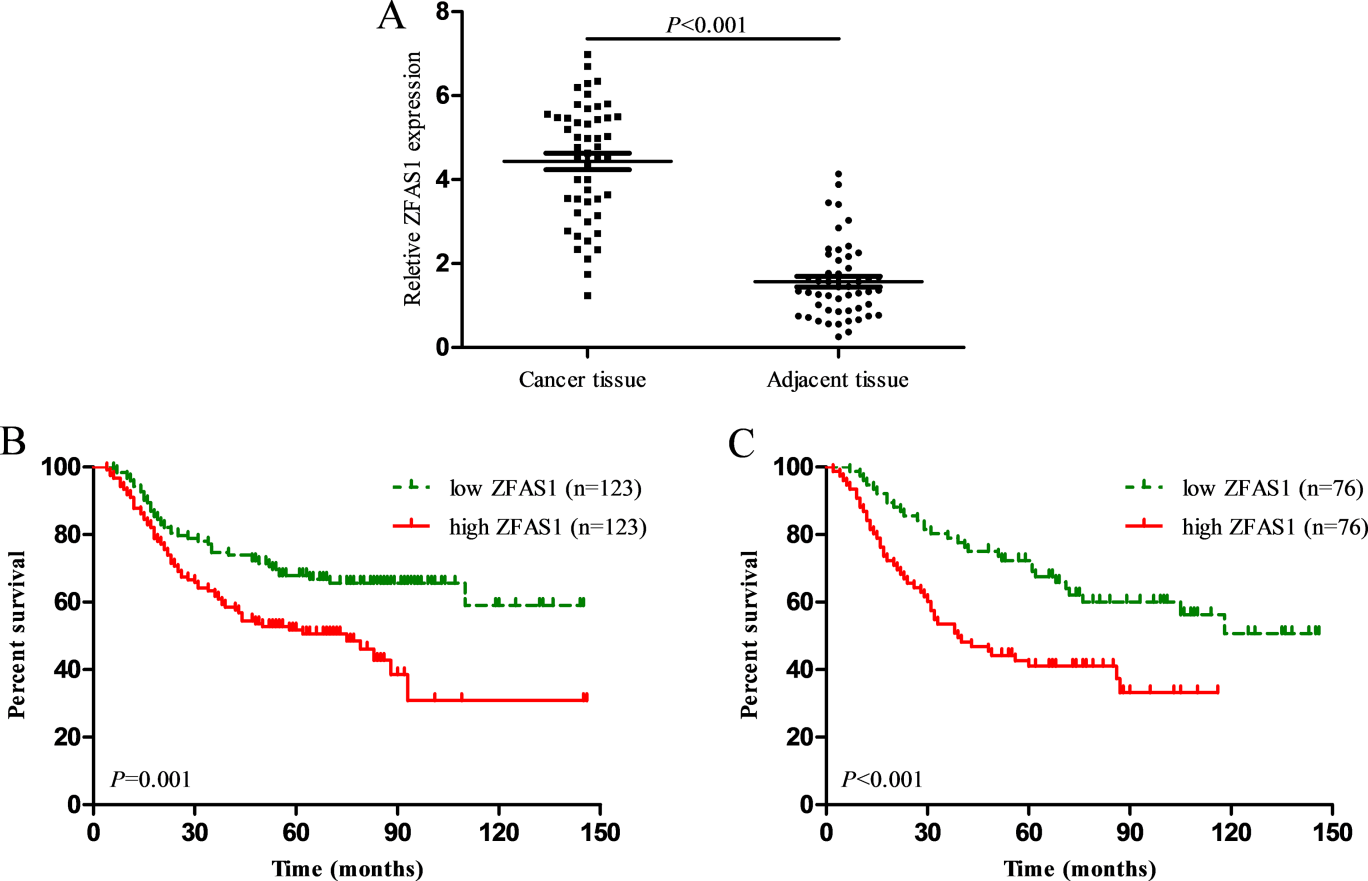
(**A**) LncRNA ZFAS1 expression was significantly higher in ESCC tissues compared with the corresponding adjacent tissues (*P* < 0.001); (**B**) ESCC patients with high ZFAS1 expression had a significantly shorter overall survival than those with low ZFAS1 expression in primary cohort (*P* = 0.001); (**C**) ESCC patients with high ZFAS1 expression had a significantly shorter overall survival than those with low ZFAS1 expression in validation cohort (*P* < 0.010).

To determine whether ZFAS1 expression was different between tumor tissues and adjacent non-cancerous tissues, we examined 50 pairs of human ESCC samples from the Third People's Hospital of Yancheng and analyzed lncRNA ZFAS1 expression by qRT-PCR. The relative expression of ZFAS1 in the cancerous tissues normalized to GAPDH was 4.44 ± 1.40 (mean ± SD), whereas the relative expression of ZFAS1 in adjacent normal tissues was 1.57 ± 0.42. The results indicated that ZFAS1 expression was significantly up-regulated in ESCC tissues compared with the corresponding adjacent tissues (*P* < 0.001, Figure 1A).

Next, the median ZFAS1 expression was used as a cutoff value to divide patients into two groups based on high ZFAS1 expression or low ZFAS1 expression. As shown in Table 1, we found that ZFAS1 expression positively correlated with histological grade (*P* = 0.010) in the primary cohort. However, there were no significant correlations between ZFAS1 expression and other clinicopathological factors, such as sex, age, tumor location, examined lymph nodes, T stage or TNM stage in the primary cohort. Similar results were observed in the validation cohort.

**Table 1:** Correlation between lncRNA ZFAS1 expression and clinicopathological factors of ESCC patients

Clinical parameter	Primary cohort				Validation cohort			
	ZFAS1 Low (123)	ZFAS1 High (123)	χ^2^	*P*	ZFAS1 Low (76)	ZFAS1 High (76)	χ^2^	*P*
Sex			0.02	0.881			2.11	0.147
Male	94	93			51	59		
Female	29	30			25	17		
Age			0.02	0.898			0.68	0.410
< 60	66	65			47	42		
≥ 60	57	58			29	34		
Histological grade			9.25	0.010*			8.68	0.013*
Well differentiated	13	5			9	1		
Moderately differentiated	70	57			38	34		
undifferentiated	40	61			29	41		
Tumor location			2.50	0.286			1.27	0.529
Upper	8	7			9	5		
Middle	75	64			60	64		
Lower	40	52			7	7		
Examined lymph nodes			0.07	0.798			1.03	0.309
< 15	67	69			52	46		
≥ 15	56	54			24	30		
T stage			0.83	0.662			3.06	0.216
T1	41	35			22	15		
T2	34	39			27	24		
T3	48	49			27	37		
8th AJCC stage			1.20	0.273			0.79	0.374
I	44	35			25	20		
II	80	88			51	56		

### High ZFAS1 expression in ESCC predicts poor patient survival

To evaluate the prognostic value of ZFAS1, survival analysis was conducted in 398 ESCC patients without lymph node metastasis. Among them, 246 patients were in the primary cohort, and 152 patients were in the validation cohort. In the primary cohort, the median follow-up time was 57.2 months. The median OS time was 98.9 months, and the 3- and 5-year OS rates were 68.3% and 59.7%, respectively. In the validation cohort, the median follow-up time was 57.2 months. The median OS time was 88.3 months, and the 3- and 5-year OS rates were 66.3% and 58.9%, respectively. As shown in Figure 1B, patients in the high ZFAS1 group had a worse overall survival (OS) compared with those in the low ZFAS1 group for the primary cohort (*P* = 0.001). Univariate analysis showed that poorly or undifferentiated, advanced T stage, and high ZFAS1 expression were poor prognostic factors in the primary cohort (*P* < 0.01, Table 2). Multivariate analyses showed that ZFAS1 expression was an independent prognostic factor in the primary cohort (HR = 1.59, 95% CI 1.07–2.36, *P* = 0.022) (Table 2). In the validation cohort, high ZFAS1 expression also correlated with poor OS, and ZFAS1 expression was also found to be an independent prognostic factor. (*P* < 0.001, Figure 1C).

**Table 2:** Univariate and multivariate cox regression analyses for overall survival in patients with ESCC in primary cohort

Variables	Univariate analysis		Multivariate analysis	
	HR (95%CI)	*P* value	HR (95%CI)	*P* value
Sex				
Male vs. Female	0.69 (0.43–1.13)	0.141		
Age				
≥ 60 years vs. < 60 years	0.94 (0.64–1.37)	0.737		
Histological grade		0.002*		0.011*
Well differentiated	Ref.		Ref.	
Moderately differentiated	2.90 (0.90–9.31)	0.074	1.77 (0.54–5.78)	0.346
Poorly or not differentiated	4.87 (1.52–15.59)	0.008*	2.99 (0.92–9.75)	0.069
Tumor location		0.900		
Upper	Ref.			
Middle	0.89 (0.41–1.96)	0.778		
Lower	0.84 (0.38–1.88)	0.672		
Examined lymph nodes				
≥ 15 vs. < 15	0.81 (0.55–1.19)	0.285		
T stage		< 0.001*		< 0.001*
T1	Ref.		Ref.	
T2	2.12 (1.17–3.85)	0.013*	2.01 (1.10–3.68)	0.023*
T3	3.77 (2.20–6.47)	< 0.001*	3.46 (2.00–5.98)	< 0.001*
ZFAS1				
Low vs. High	1.89 (1.28–2.79)	0.002*	1.59 (1.07–2.36)	0.022*

**Table 3:** Univariate and multivariate cox regression analyses for overall survival in patients with ESCC in validation cohort

Variables	Univariate analysis		Multivariate analysis	
	HR (95%CI)	*P* value	HR (95%CI)	*P* value
Sex				
Male vs. Female	0.79 (0.46–1.36)	0.394		
Age				
≥ 60 years vs. < 60 years	1.51 (0.98–2.48)	0.053		
Histological grade		0.004*		0.025*
Well differentiated	Ref.		Ref.	
Moderately differentiated	5.61 (0.77–41.21)	0.090	2.59 (0.34–19.81)	0.359
Poorly or not differentiated	9.87 (1.42–74.72)	0.021*	4.65 (0.61–35.35)	0.069
Tumor location		0.753		
Upper	Ref.			
Middle	1.00 (0.46–2.18)	0.997		
Lower	0.70 (0.22–2.23)	0.551		
Examined lymph nodes				
≥ 15 vs. < 15	1.01 (0.63–1.61)	0.982		
T stage		< 0.001*		0.005*
T1	Ref.		Ref.	
T2	2.28 (1.05–4.93)	0.037*	2.20 (1.00–4.81)	0.049*
T3	4.08 (1.99–8.37)	< 0.001*	3.25 (1.56–6.75)	0.002*
ZFAS1				
Low vs. High	2.20 (1.38–3.52)	0.001*	1.81 (1.13–2.91)	0.014*

### Nomogram development

**Figure 2:**
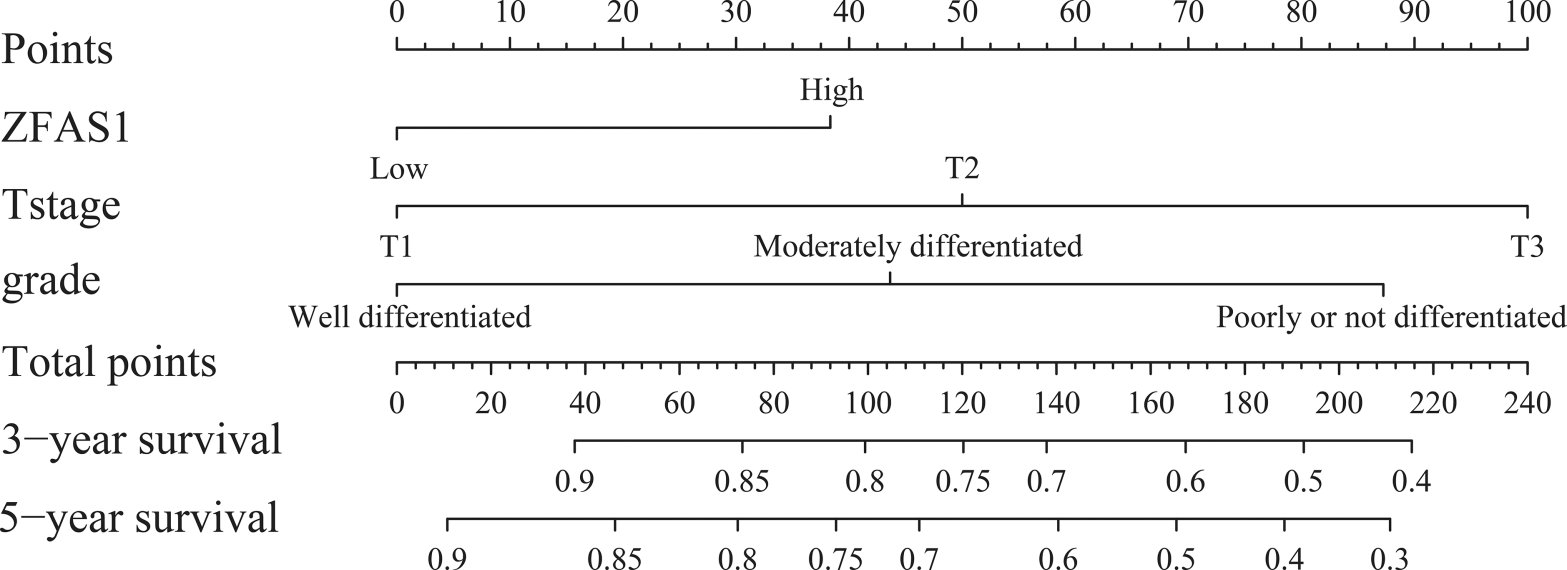
Evaluation of nomogram integrated ZFAS1 and clinicopathological factors in the lymph node-negative ESCC patients To use the nomogram, the value attributed to an individual patient is located on each variable axis, and a line is drawn upwards to determine the number of points received for each variable value. The sum of these numbers is located on the total points axis, and a line is drawn downward to the survival axis to determine the likelihood of 3- or 5-year survival.

**Figure 3:**
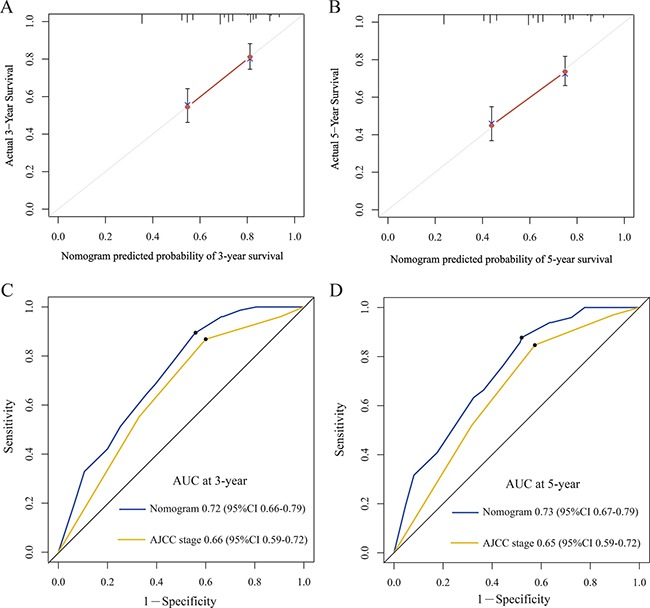
The calibration curve for predicting patient survival at 3-year (**A**) and 5-year (**B**) in the primary cohort. Time-dependent receiver operating characteristic (ROC) curves by nomogram and 8^th^ AJCC-TNM staging system for 3-year (**C**) and 5-year (**D**) OS in the primary cohort.

**Figure 4:**
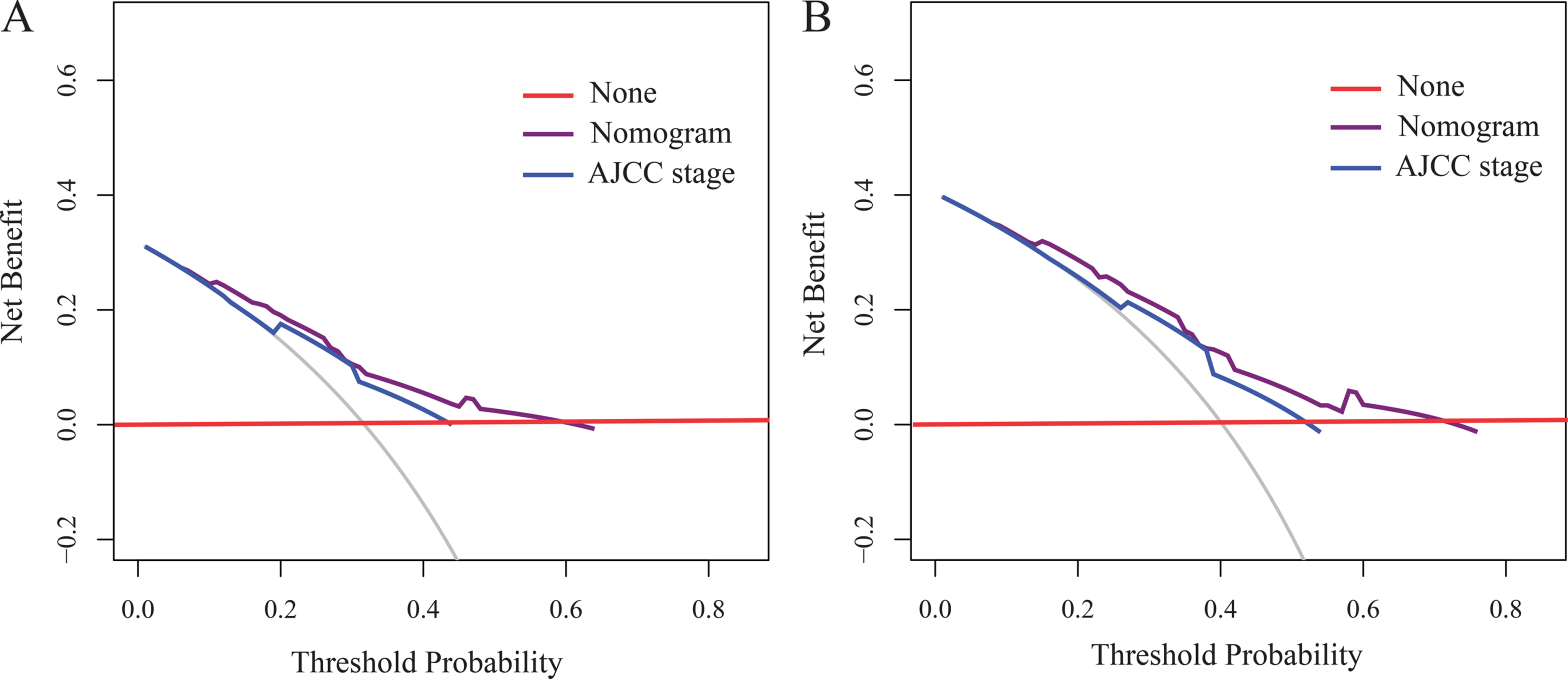
Decision curve analyses by nomogram and 8th AJCC-TNM staging system for 3-year (**A**) and 5-year (**B**) OS in the primary cohort.

For the development of the nomograms, data from 246 patients from the Third People's Hospital of Yancheng were used. Multivariate analyses demonstrated that histological grade, T stage and ZFAS1 expression were independent risk factors for OS in the primary cohort (Table 2). Backward stepwise selection with the AIC was used to build a predictive nomogram. Finally, the nomogram that integrated histological grade, T stage and ZFAS1 expression was used to predict 3- and 5-year ESCC patient survival (Figure 2). The calibration plots for the probability of 3- or 5-year survival showed good agreement between the observed outcomes and the prediction made by the nomogram (Figure 2A and 2B). The discrimination ability of the nomogram for OS was assessed by the C-statistic. The concordance index (C-index) of the nomogram (C-index = 0.71, 95% CI 0.66–0.76) was significantly higher than TNM stage (C-index = 0.63, 95% CI 0.59–0.67) (*P* < 0.001). Receiver operating characteristics (ROC) curve analysis indicated that the nomogram also displayed better area under curve (AUC) values than TNM stage at 3 and 5 years (Figure 3C and 3D). In the decision curve analysis, the nomogram demonstrated higher net benefit gains throughout the entire range of threshold probabilities for survival after 3 or 5 years compared with TNM stage (Figure 4A and 4B).

### External validation of the nomogram

**Figure 5:**
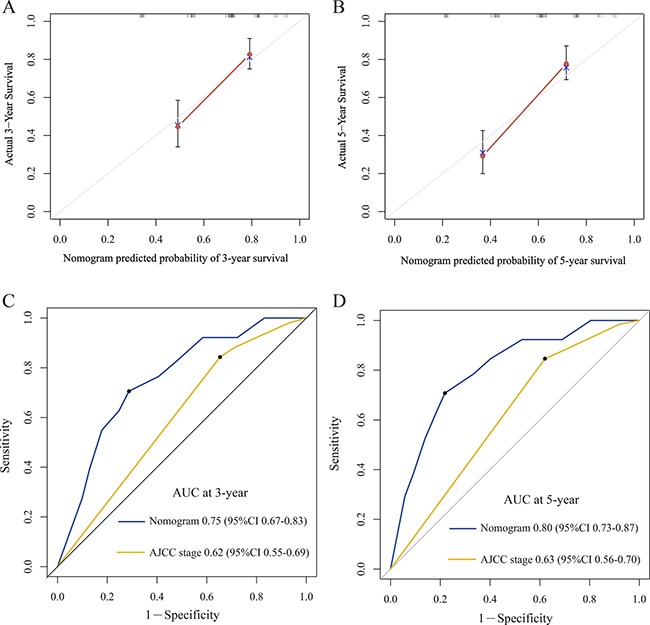
The calibration curve for predicting patient survival at 3-year (**A**) and 5-year (**B**) in the validation cohort. Time-dependent receiver operating characteristic (ROC) curves by nomogram and 8th AJCC-TNM staging system for 3-year (**C**) and 5-year (**D**) OS in the validation cohort.

Next, to validate the nomogram, an independent validation cohort of 152 patients from the Yancheng City No.1 People's Hospital was evaluated. The calibration curves using this validation cohort also showed good correlation between the actual outcome and the predicted outcome (Figure 5A and 5B). The C-index of the nomogram for predicting OS was 0.74 (95% CI 0.70 to 0.80), which was significantly better than TNM stage (C-index = 0.63, 95% CI 0.58–0.69) (*P* < 0.001). The ROC curve also displayed better AUC values than TNM stage at 3 and 5 years (Figure 5C and 5D). These results demonstrated that our nomogram performs better at predicting OS than TNM stage in ESCC patients without lymph node metastasis.

## DISCUSSION

The earliest literature reports on ZFAS1 were seen in 2011 [9]. Askarian-Amiri et al. found that ZFAS1 is highly expressed in normal breast tissue and down-regulated in breast cancer tissue, and the knockdown of ZFAS1 in an epithelial cell line of breast cancer promoted cell proliferation, which suggested that ZFAS1 might be a tumor suppressor gene in breast cancer [9]. Then, Tao et al. found high expression of ZFAS1 in liver cancer, which was associated with metastasis and poor prognosis [10]. Mechanistically, ZFAS1 was found to act as a competing endogenous RNA (ceRNA) in liver cancer by binding to miR-150 and inhibiting the tumor suppressor function of miR-150 [10]. Recent research has also found that ZFAS1 acts as an oncogene in colorectal cancer. The expression of ZFAS1 in colorectal cancer was high and significantly related to lymphatic metastasis. Therefore, ZFAS1 is an independent prognostic factor of recurrence and death for colorectal cancer patients [14], and down-regulation of ZFAS1 could inhibit migration and invasion of intestinal cancer cells [12]. In addition, ZFAS1 promoted cell cycle progression and inhibited apoptosis by inducing P53 instability and interacting with the CDK1/cyclin B1 complexes [12]. The expression of ZFAS1 in gastric cancer tissue was also found to be significantly higher than that of para-carcinoma tissue, and its high expression was significantly related to poor overall patient survival [13]. *In vitro* experiments also showed that gastric cancer cell proliferation decreased and apoptosis increased after ZFAS1 knockdown [13]. Mechanistically, ZFAS1 was found to promote gastric cancer cell proliferation by inhibiting KLF2 and NKD2 expression [13]. Experiments *in vivo* also showed that knockdown of ZFAS1 inhibited the tumorigenic ability of gastric cancer cells, and mechanistic experiments validated that ZFAS1 could promote the proliferation of gastric cancer cells by inhibiting the expression of KLF2 and NKD2 [13]. In addition, Zhou et al. validated that ZFAS1 was highly expressed in gastric cancer tissue and plasma and that the higher the ZFAS1 expression, the stronger the EMT potential of circulating tumor cells [15]. Pan et al. also provided further evidence that ZFAS can promote the proliferation and metastasis of gastric cancer cells by exosomes [21]. ZFAS1 promoted the increased expression of SP1 in ovarian cancer by competitive antagonism against miR-150 to enhance the ability of cell proliferation and chemotherapy resistance for ovarian cancer cells [19]. The expression of ZFAS1 has also been reported to be high, and correlates significantly with poor prognosis in glioma tissues and cells [18]. In addition, *in vitro* experiments demonstrated that ZFAS1 knockdown in glioma cells inhibited the proliferation and invasion of glioma cells. EMT and Notch signaling pathways in glioma cells were inactivated after ZFAS1 knockdown. ZFAS1 expression was also significantly increased [18]. In lung cancer, high ZFAS1 expression was significantly related to the poor prognosis [17]. Therefore, ZFAS1 expression in a variety of tumors was up-regulated and significantly correlated with poor prognosis, except in breast cancer.

Although ZFAS1 has been widely reported to play an important role in the development and progression of many types of cancer, the expression and prognostic value of ZFAS1 in ESCC are still not clear. Our work demonstrated for the first time that the expression of ZFAS1 in ESCC was significantly higher than that in normal para-carcinoma tissue and that ZFAS1 expression is closely related to the ESCC histological grade. Moreover, our data indicated that patients with high ZFAS1 expression have a significantly shorter OS than those with low ZFAS1 expression. Multivariate analysis showed that ZFAS1 was an independent prognostic factor in ESCC patients. Previous studies have shown that nomograms can predict the tumor prognosis more accurately than the traditional AJCC TNM in esophageal cancer [23–25]. In this study, we integrated clinicopathological factors and ZFAS1 expression to develop and validate a new prognostic nomogram that could predict the prognosis of ESCC patients better than the traditional staging system. The prediction accuracy of the nomogram for survival was as high as 0.72 C indexes. The prediction results were significantly better than the 8th TNM staging system in the prediction of the survival time. Compared with the TNM staging system, the ROC curve was more sensitive for predicting 3- and 5-year overall survival with specificity. Importantly, these results were confirmed using an independent test group consisting of external data. The calibration drawing lines of initial queue and verification queue also revealed that the predicted survival probability was highly consistent with the actual one. The analysis of the decision-making curve showed that our model was better than the TNM staging system with regards to predicting survival. Therefore, our nomogram reliably predicted patient survival for patients with resectable ESCC and may contribute to determining personalized therapeutics in the future.

While our nomogram accurately predicted the postoperative survival of lymph node-negative ESCC patients, there were still many limitations in our study. First, our nomogram only contained the lncRNA ZFAS1, but other lncRNAs, such as HOTAIR, H19 and ANRIL, may also need to be considered for improving the prediction of the patients. Second, our study was a retrospective study, which might result in a selection bias in the collection of data. Third, the esophageal cancer group for our nomogram was limited because we only included lymph node-negative ESCC patients without preoperative chemoradiotherapy.

In conclusion, lncRNA ZFAS1 expression was up-regulated in ESCC and its over-expression was associated with a poor prognosis. Our proposed nomogram integrated clinicopathological factors, and ZFAS1 accurately predicted the prognosis of lymph node-negative ESCC patients without preoperative chemoradiotherapy. We believe that our nomogram is a reliable and useful tool, which can help in therapeutic decision-making and individualized patient counseling.

## MATERIALS AND METHODS

### Patients

The patients in this study were divided into two groups: the primary cohort and the validation cohort. The primary cohort included 246 ESCC patients who had undergone radical esophagectomy in the Third People's Hospital of Yancheng from January 2002 to December 2012. The validation cohort included 152 ESCC patients who had undergone a radical esophagectomy in Yancheng City No.1 People's Hospital from January 2002 to December 2012. The inclusion criteria were as follows: ESCC, R0 resection, no other malignant tumor, no lymph node metastasis, no distant metastasis, no radiotherapy and/or chemotherapy before or after surgery. Follow-up was conducted as described previously [26]. The research agreement was signed according to the guidelines formulated by the Declaration of Helsinki after the approval of the Ethics Committee of the Third People's Hospital of Yanchen.

### Quantitative reverse transcription-polymerase chain reaction analysis (qRT-PCR)

50 paired fresh surgically resected ESCC tumor tissues and adjacent non-tumor tissues were collected from the Third People's Hospital of Yanchen between January 2014 and December 2015. The specimens were immediately frozen in liquid nitrogen and stored at –80°C until use. Total RNA from tissues was extracted using TRIzol reagent (Invitrogen, Carlsbad, CA, USA) and reverse transcribed into cDNA according to the manufacturer's instructions. The following gene-specific primers were used in this study: forward, 5′-ACGTGCAGAC ATCTACAACCT-3′ and reverse 5′-TACTTCCAACAC CCGCAT-3′ for lncRNA ZFAS1; forward, 5′-GGTCTCC TCTGACTTCA-3′ and reverse 5′- GTGAGGGTCTCTC TCTTCCT-3′ for GAPDH. The relative expression level of lncRNA ZFAS1 was normalized to that of the internal control GAPDH using the comparative delta CT (2^-ΔΔCt^) method.

### Statistical analysis

Statistical analysis was conducted using SPSS 17.0 (SPSS, Chicago, IL) and R software version 3.2.5 (http://www.r-project.org/) with Hmisc, RMS, and the survival ROC statistical packages. The survival curve was drawn according to the Kaplan-Meier method, and compared by log-rank test. All variables that reached the statistical significance of *P* < 0.05 in univariate analysis were included in the multivariate Cox proportional hazard model. The nomogram was formulated according to the results of the multivariate analysis. A backward selection process was conducted stepwise for the final model selection according to Akaike information criterion. The discrimination and calibration of these models were assessed at the same time to assess the efficiency of the nomogram. Time-dependent ROC curves and C-index were used to compare the ability to distinguish for different models on the overall survival. A total of 1,000 repeated sampling inspections from the sample were conducted with repeating the estimation process to finally obtain the confidence interval (CI). The larger the C index, the more accurate the prognosis prediction. The clinical application of the model was evaluated and predicted through quantitative analysis of net benefit by decision-making curve. For the nomogram external validation, the total scores for each patient in the validation queue were calculated according to the generated nomogram. Then, Cox regression analysis of this queue was conducted with the total scores as a prognostic factor. Finally, the C index and the calibration curve were deduced according to the regression analysis. The previous guidance was used to build and validate the nomogram. *P* < 0.05 was regarded as statistically significant unless otherwise specified.
